# An Early Increase in IL-10 and TNF-α Levels Following Atezolizumab Plus Bevacizumab Treatment Predicts Survival in Advanced Hepatocellular Carcinoma Patients: A Prospective Cohort Study

**DOI:** 10.3390/cancers16203543

**Published:** 2024-10-21

**Authors:** Soon Kyu Lee, Soon Woo Nam, Ji Won Han, Jung Hyun Kwon

**Affiliations:** 1 Division of Gastroenterology and Hepatology, Department of Internal Medicine, Incheon St. Mary’s Hospital, College of Medicine, The Catholic University of Korea, Seoul 06591, Republic of Korea; blackiqq@catholic.ac.kr (S.K.L.); drswnam@catholic.ac.kr (S.W.N.); 2 Division of Gastroenterology and Hepatology, Department of Internal Medicine, Seoul St. Mary’s Hospital, College of Medicine, The Catholic University of Korea, Seoul 06591, Republic of Korea; tmznjf@catholic.ac.kr

**Keywords:** hepatocellular carcinoma, biomarker, cytokine, immune checkpoint inhibitor, outcome

## Abstract

**Background/Objectives**: Reliable biomarkers for predicting outcomes in hepatocellular carcinoma (HCC) treated with atezolizumab plus bevacizumab (Ate/Bev) are still lacking. Cytokines, which play a crucial role in immune regulation and HCC progression, have potential as predictive markers, but data supporting their use are limited. This study aimed to evaluate the impact of early changes in cytokine levels on the clinical outcomes of advanced HCC patients. **Methods**: We prospectively enrolled 32 advanced HCC patients, collecting blood samples before the first and second Ate/Bev treatments. These samples were analyzed for IL-2, IL-6, IL-10, IL-12, IL-17, IFN-γ, and TNF-α levels to assess changes post-treatment. The primary outcome was overall survival, with a secondary focus on progression-free survival (PFS) at 6 months. **Results**: The mean age of the participants was 64.2 years, with the majority being male (93.8%). Patients showing increased IL-10, IL-17, and TNF-α levels had significantly better survival (*p* < 0.05) and marginally improved PFS compared to those with decreased cytokine levels. Interestingly, a positive correlation was noted between changes in IL-10 and TNF-α levels (*p* = 0.009). Furthermore, a multivariable analysis revealed that increased levels of IL-10 and TNF-α were significant predictors of enhanced survival (hazard ratio, 0.07; 95% confidence interval, 0.01–0.46; *p* = 0.005). **Conclusions**: An early increases in IL-10 and TNF-α after Ate/Bev treatment may serve as effective biomarkers for clinical outcomes in advanced HCC patients.

## Simple Summary

Due to a shortage of reliable biomarkers for predicting outcomes in hepatocellular carcinoma (HCC) following treatment with atezolizumab plus bevacizumab (Ate/Bev), we assessed the impact of early changes in cytokine levels on the clinical outcomes of advanced HCC patients. We prospectively enrolled 32 patients, analyzing changes in IL-2, IL-6, IL-10, IL-12, IL-17, IFN-γ, and TNF-α levels from blood samples collected before the first and second Ate/Bev treatments. Patients with increased IL-10, IL-17, and TNF-α levels demonstrated significantly better survival and marginally improved progression-free survival compared to those with decreased cytokine levels. Additionally, a positive correlation was found between changes in IL-10 and TNF-α levels. Multivariable analysis confirmed that increases in these cytokines are significant predictors of improved survival (*p* = 0.005). Early increases in IL-10 and TNF-α levels post-Ate/Bev treatment may thus serve as effective biomarkers for clinical outcomes in advanced HCC patients.

## 1. Introduction

In the treatment of advanced hepatocellular carcinoma (HCC), there has been a recent revolutionary advance represented as the superior efficacy of atezolizumab plus bevacizumab (Ate/Bev) treatment over sorafenib [1,2]. Indeed, Ate/Bev treatment showed significantly better survival and progression-free survival (PFS) compared to those treated with sorafenib, which was the previous first-line therapy for advanced HCC [1,2]. With these promising results, Ate/Bev treatment has become a first-line therapy in patients with advanced HCC [3].

In terms of mechanism, immune checkpoint inhibitors (ICIs), such as atezolizumab, durvalumab, nivolumab, and pembrolizumab, inhibit programmed cell death protein-1 (PD-1) or programmed death-ligand 1 (PD-L1) in restoring the function of effector CD8+ T cells (Teffs) in patients with HCC [4]. Moreover, the addition of anti-Vascular endothelial growth factor (VEGF) drugs, including bevacizumab, has a synergistic effect by reducing the immunosuppressive effects of VEGF on immune cells and the normalization of the vessels [4,5]. Despite these mechanisms, the response to ICIs is quite heterogeneous, raising the need for biomarkers in predicting treatment response.

Recently, it has been reported that a high density of intra-tumoral CD8+ T cells was associated with better outcomes, while a high regulatory T cell (Tregs)-to-Teffs ratio was associated with a reduced clinical benefit in HCC patients treated with Ate/Bev [6]. Along with these immune cells, several cytokines are also involved and contribute to HCC formation and progression, such as transforming growth factor-β (TGF- β) for preventing cancer cell apoptosis and interleukin-6 (IL-6) for promoting cell proliferation [7]. Recent studies have highlighted the significant roles of cytokines such as IL-10, IL-17, and TNF-α in immune regulation and tumor progression. IL-10 is an anti-inflammatory cytokine known to modulate immune responses and maintain immune homeostasis, while IL-17 is involved in promoting pro-inflammatory responses, contributing to tumor development. TNF-α, on the other hand, has a dual role in tumor progression by not only promoting inflammation but also inducing apoptosis in tumor cells [6,7]. However, there have been limited data on investigating cytokines as potential biomarkers for predicting outcomes following Ate/Bev treatment, especially in evaluating the impact of their early changes after Ate/Bev therapy. Therefore, understanding the changes in these cytokine levels after Ate/Bev treatment can provide valuable insights into their roles as potential biomarkers for predicting clinical outcomes in HCC patients.

Herein, we prospectively evaluate the impact of early changes in cytokine levels following Ate/Bev therapy on survival prediction in patients with advanced HCC. Additionally, we examined the treatment response and PFS in relation to those changes, as well as the relationship among cytokine levels themselves.

## 2. Materials and Methods

### 2.1. Patients

**Figure 1:**
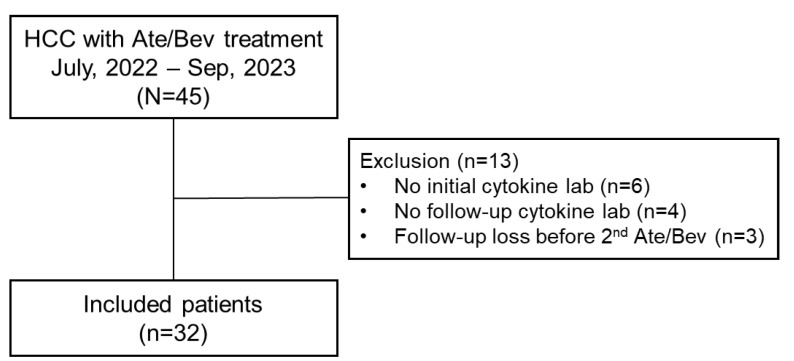
Study flow chart. HCC, hepatocellular carcinoma; Ate/Bev, atezolizumab plus bevacizumab.

We prospectively enrolled 45 consecutive patients with advanced HCC who were undergoing treatment with Ate/Bev therapy between July 2022 and September 2023. Ate/Bev treatment was administered intravenously with a dose of 1200 mg of atezolizumab plus 15 mg/kg of bevacizumab every 3 weeks [2]. Following enrollment, initial blood samples were collected from each patient before their first Ate/Bev treatment. Subsequently, follow-up blood samples were taken one day prior to their second Ate/Bev treatment, scheduled 3 weeks after the initial treatment. Out of 45 patients, 13 patients were excluded due to the following reasons: patients without initial cytokine data (*n* = 6), patients without follow-up cytokine data (*n* = 4), and follow-up loss before the 2nd Ate/Bev treatment. Finally, 32 patients were included in the analysis (Figure 1). This study was performed in accordance with the Declaration of Helsinki and was approved by the Institutional Review Board of the Incheon St. Mary’s Hospital, The Catholic University of Korea (OC22TISI0039).

### 2.2. Clinical and Laboratory Data

At the time of starting Ate/Bev treatment, baseline clinical and laboratory data, including age, sex, cause of HCC, platelet count, total bilirubin, albumin, aspartate transaminase (AST), alanine transaminase (ALT), albumin, international normalized ratio, creatinine, Child–Pugh score, alpha-fetoprotein (AFP), and protein induced by vitamin K absence-II (PIVKA-II) were checked. Furthermore, characteristics of HCC in the included patients, such as the tumor size, number of tumors, presence of portal vein tumor thrombosis (PVTT), and extrahepatic metastasis, were also checked at the start time of Ate/Bev treatment.

### 2.3. Cytokine Analysis

Using collected blood samples, cytokine concentrations were quantified using the MILLIPLEX MAP human cytokine/chemokine panel (Millipore, Darmstadt, Germany) on the Luminex 200 instrument (Luminex, Austin, TX, USA). These samples were analyzed to measure the levels of IL-2, IL-6, IL-10, IL-12, IL-17, IFN-γ, and TNF-α and to calculate the changes in these levels before and after the 1st Ate/Bev treatment.

### 2.4. Outcomes Assessment

The primary outcome was overall survival (OS) according to the changes in the cytokine levels. We also evaluated the factors for predicting OS, including the changes in cytokine levels. The OS was determined as the time length from the time of the initiation of Ate/Bev treatment to either the last follow-up or death. Secondary outcomes were progression-free survival (PFS) at 6 months and treatment response according to the cytokine changes. PFS at 6 months was referred to as the time interval between the first treatment time and progression time. When the impact of cytokine level changes on clinical outcomes was analyzed, including the OS and PFS at 6 months, the changes were categorized into two groups based on their median values. Treatment responses were evaluated by multiphase liver dynamic CT or MRI every 2–3 months following Response Evaluation Criteria in Solid Tumors 1.1 (RECIST 1.1) [8].

### 2.5. Statistical Analysis

The patient’s baseline characteristics were described as a mean ± standard deviation or median (interquartile range [IQR] or range) for quantitative variables and as counts (%) for categorical variables, appropriately. For group comparisons, the Student’s *t*-test or Mann–Whitney U test was used for continuous variables, while the chi-square test or Fisher’s exact test was conducted for categorical variables, as appropriate. We performed Kaplan–Meier analysis for analyzing OS and PFS at 6 months. Cox-regression analysis was also conducted to identify the risk factors for predicting OS. *p*-values < 0.05 were considered as significant. The statistical analyses performed in this study were conducted by R software (version 4.3.3).

## 3. Results

### 3.1. Baseline Characteristics

The baseline characteristics of the participants are described in Table 1. The mean age of the included patients (*n* = 32) was 64.2 years, and 93.8% (*n* = 30) of the included patients were male. The major cause of HCC was related to an HBV infection (*n* = 24, 75%). All patients had Child–Pugh class A with median levels of AFP and PIVKA at 77.9 ng/mL and 1085.2 mAU/mL, respectively. The median tumor size was 5.8 cm, and 28 (87.5%) patients had multiple intrahepatic HCC. About 70% of patients (*n* = 22) had PVTT, while 17 (53.1%) had extrahepatic metastasis.

### 3.2. OS According to Changes in Cytokine Levels

**Figure 2:**
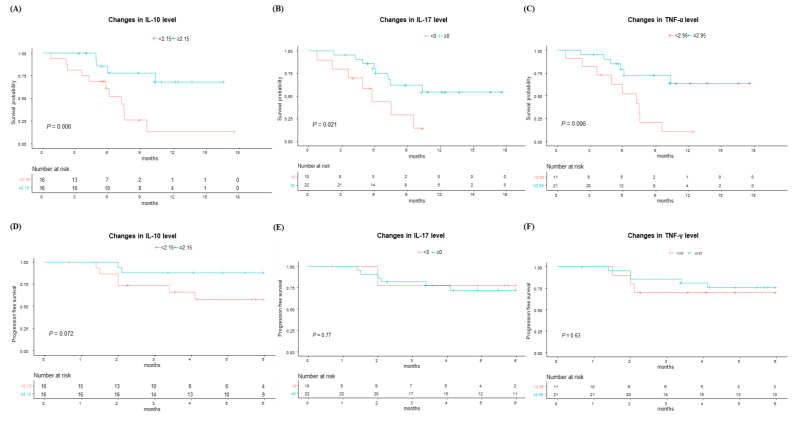
(**A**–**C**) Overall survival and (**D**–**F**) progression-free survival at 6 months according to changes in cytokine levels before and after 1st atezolizumab/bevacizumab treatment, including IL-10, IL-17, and TNF-α, based on their median changes in levels.

First, we evaluated the OS according to the changes in each cytokine level before and after the first Ate/Bev treatment, including IL-2, IL-6, IL-10, IL-17, IL-12, TNF-α, and IFN-γ. Interestingly, as depicted in Figure 2A–C, patients who showed an increase in IL-10, IL-17, and TNF-α levels demonstrated significantly better survival compared to those with decreased levels in these cytokines (*p* < 0.05 for all). Meanwhile, changes in other cytokines, including IL-2, IL-6, IL-12, and IFN-γ, did not show a significant impact on survival outcomes (Supplementary Figure S1). Additionally, when evaluating the impact of baseline cytokine levels, no significant differences were observed in the OS (Supplementary Figure S2).

### 3.3. PFS at 6 Months and Treatment Response According to Changes in Cytokine Levels

Next, we evaluated the PFS at 6 months according to the changes in cytokine levels before and after the first Ate/Bev treatment (Figure 2D–F and Supplementary Figure S3). The patients with increased IL-10 exhibited marginally better PFS at 6 months compared to those without such increases. However, other cytokines did not exhibit significant differences in PFS at 6 months. Moreover, there were no significant differences in the PFS at 6 months according to the baseline cytokine levels (Supplementary Figure S2).

**Figure 3:**
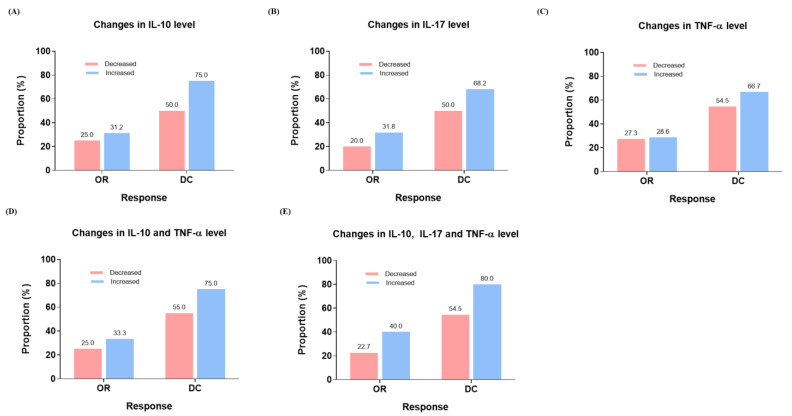
Treatment response to changes in cytokine levels before and after 1st atezolizumab/bevacizumab treatment, including (**A**) IL-10, (**B**) IL-17, (**C**) TNF-α, (**D**) IL-10 and TNF-α, and (**E**) IL-10, IL-17, and TNF-α, based on their median changes in levels. OR, objective response; DC, disease control.

Regarding treatment response, patients who experienced an increase in IL-10, IL-17, and TNF-α levels also demonstrated marginally higher treatment responses compared to those without such an increase (Figure 3A–C). Furthermore, patients with increased IL-10 and TNF-α (Figure 3D) and IL-10, IL-17, and TNF-α (Figure 3E) still showed marginally better treatment response than patients with a decrease in those cytokines.

### 3.4. Correlation Between Cytokine Levels

**Figure 4:**
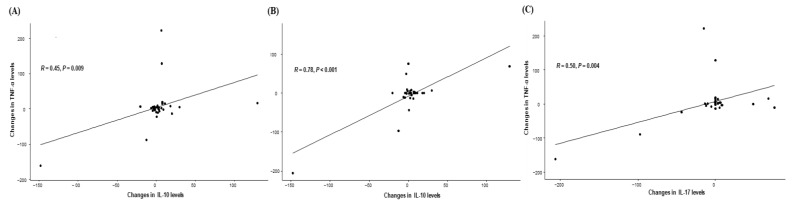
The correlation between the changes in cytokine levels before and after 1st atezolizumab/bevacizumab treatment including (**A**) IL-10 and IL-17, (**B**) IL-10 and TNF-α, and (**C**) IL-17 and TNF-α.

Based on the significant impact of changes in IL-10, IL-17, and TNF-α on clinical outcomes, we next evaluated the correlation between those cytokines. Interestingly, a significant positive correlation was observed between the changes in IL-10 and TNF-α levels (*p* = 0.009), IL-10 and IL-17 (*p* < 0.001), and IL-17 and TNF-α (*p* = 0.004), suggesting their possible interaction and simultaneous impact of clinical outcomes (Figure 4).

### 3.5. OS and PFS at 6 Months According to Combination of Cytokine Level Changes

**Figure 5:**
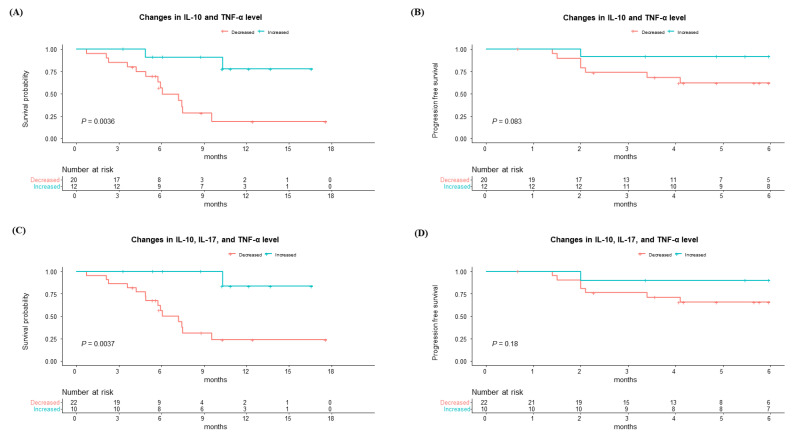
Overall survival and progression-free survival at 6 months according to changes in cytokine levels before and after atezolizumab/bevacizumab treatment, including (**A**,**B**) IL-10 and TNF-α and (**C**,**D**) IL-10, IL-17, and TNF-α, based on their median changes in levels.

Considering these positive correlations between IL-10, IL-17, and TNF-α, we further investigated the OS and PFS at 6 months according to those cytokines. Patients who experienced an increase in IL-10 and TNF-α exhibited significantly better survival alongside marginally better PFS at 6 months compared to those without such an increase (Figure 5A,B). Moreover, patients who had an increase in IL-10, IL-17, and TNF-α also showed higher survival rates with a tendency to better PFS at 6 months than those with a decrease (Figure 5C,D).

### 3.6. Predicting Factors for OS Including Changes in Cytokine Levels

Finally, we evaluated the factors for predicting OS in patients with Ate/Bev treatment, including the changes in cytokine levels before and after the first Ate/Bev treatment (Table 2). In univariate and multivariable analysis, intrahepatic tumor size was a significant predicting factor for OS. Along with this factor, early increases in each IL-10 (hazard ratio [HR], 0.19; *p* = 0.014) and in TNF-α levels (HR, 0.23; *p* = 0.009) are also significant factors for improving survival in patients with Ate/Bev therapy, while changes in IL-17 were not a significant factor. When combining those cytokines, an increase in IL-10, IL-17, and TNF-α levels after the first Ate/Bev therapy was a significant factor for survival (HR, 0.09; *p* = 0.024). Furthermore, an increase in both IL-10 and TNF-α levels after the first Ate/Bev therapy emerged as the best predictor of improved survival with the highest reduction in mortality (HR, 0.07; *p* = 0.005) in those patients.

## 4. Discussion

This prospective cohort study is the first to demonstrate the impact of an early increase in IL-10 and TNF-α after the first Ate/Bev treatment on clinical outcomes. Indeed, an increase in the IL-10 and TNF-α significantly improved OS and emerged as a significant factor for predicting OS in advanced HCC receiving Ate/Bev treatment. Furthermore, combining those cytokines showed the highest HR for predicting OS in Ate/Bev treatment, suggesting a higher predictability in survival through their combination. Taken together, our results suggest that evaluating the early changes in cytokines, especially IL-10 and TNF-α, would be an easily accessible tool for predicting clinical outcomes in HCC patients treated with Ate/Bev.

Importantly, this prospective study is the first to reveal that an early increase in IL-10 after the first Ate/Bev treatment is associated with better clinical outcomes, especially in predicting OS. IL-10, one of the major cytokines secreted by regulatory T cells and B cells, has been suggested as a possible biomarker for diagnosing HCC [9,10,11,12]. Furthermore, some studies have suggested that increased IL-10 expression was associated with poor survival in HCC [13,14]. Higher baseline IL-10 levels might also be associated with poor survival after resection [15]. However, there have been limited data on the impact of early changes in IL-10 levels on clinical outcomes, especially in patients treated with Ate/Bev therapy. A result in our study, indicating that an early increase in IL-10 was associated with better survival, might be attributable to the reflection of compensable immune modulation alongside an increase in and activation of immunosuppressive CD8^+^ T cells and immunostimulation in response to Ate/Bev treatment [16]. A positive correlation between IL-10 and TNF-α in our study also supports this scenario and the predictive role of early changes in IL-10 on survival outcomes in Ate/Bev treatment. Further in vitro and in vivo studies are required to validate our results in this study.

Indeed, an early increase in TNF-α after the first Ate/Bev treatment was also a significant prognostic factor for OS in HCC patients treated with Ate/Bev. This result is in accordance with a recent study showing an increased TNF-α with intrahepatic CD8^+^ T cells after TIGIT blockade treatment in a mouse model of HBV-related HCC [17]. Ate/Bev treatment is known to restore the function of effector CD8+ T cells, and a recent study revealed that an expression of Ki-67 and T-cell immunoreceptors with Ig and ITIM domains (TIGIT) in CD8^+^ T cells was significantly changed after Ate/Bev treatment [5,18,19,20]. Moreover, after the activation of CD8+ T cells to cytotoxic lymphocytes (CTLs), they release perforin, granzyme, and TNF to destroy HCC cells [21]. Therefore, our results demonstrate that an early increase in TNF-α after the first Ate/Bev treatment could be an important prognostic marker for survival in HCC patients undergoing this therapy, suggesting that continuing Ate/Bev therapy could be beneficial. Meanwhile, our results indicate that patients without an early increase in TNF-α may need a timelier assessment of treatment response and consider an early change of treatment plan to second-line treatment according to the treatment response.

Furthermore, our study demonstrated that patients with early increases in both IL-10 and TNF-α had the lowest risk of mortality, with an HR of 0.07 compared to those without an increase in these cytokines. Given that combining AFP and PIVKA-II may improve the diagnosis and surveillance of HCC [22], our results suggest that a combination of those cytokines may improve the prognostic power in patients with Ate/Bev treatment. Meanwhile, changes in other cytokines, including IL-6, IL-12, and IFN-γ did not show their significance in predicting survival in Ate/Bev treatment. Considering the contributing role of cytokines, such as IL-6, to HCC development [7], further studies evaluating the impact of early changes in those cytokines on clinical outcomes are warranted to validate our results. With these findings taken together, our study is the first to demonstrate the possible role of cytokines, an easy-to-access tool, as a predictive biomarker in HCC patients.

Our study has several limitations. First, it involved a small number of participants coupled with a considerable rate of exclusion in our prospective study. Second, this is a single-center study in Korea, where HBV-related HCCs are predominant. Third, although the OS was significantly better in patients with increased IL-10 and TNF-α levels, the PFS at 6 months was only marginally better. The borderline PFS results might be attributable to the limited sample size. Therefore, our results should be validated with a larger number of patients, as well as in other countries having a different predominant etiology of HCC, such as metabolic dysfunction-associated steatotic liver disease. Furthermore, additional studies are needed to evaluate changes in these cytokine levels during later treatment periods, which could further substantiate the findings of our study. Nonetheless, our study is the first prospective study in clinical practice to evaluate the possibility of early changes in cytokines as a predictive marker for clinical outcomes in patients with Ate/Bev treatment. Through detailed analysis, we demonstrated that an early increase in IL-10 and TNF-α after the first Ate/Bev treatment can predict the OS following Ate/Bev treatment.

## 5. Conclusions

In conclusion, this prospective study provides insights into the predictive role of early changes in IL-10 and TNF-α after the first Ate/Bev treatment for clinical outcomes following Ate/Bev treatment in advanced HCC patients. Our study is the first to reveal that patients with an early increase in IL-10 and TNF-α may have a better prognosis than those without such increases in IL-10 and TNF-α. These easily accessible tools may contribute to the precision medicine for HCC patients treated with Ate/Bev, suggesting that patients with early increases in those cytokines can continue therapy, while others may need early changes in treatment plans after a timely response evaluation. Further studies with a larger number of patients would be needed in the future to validate our findings.

## Figures and Tables

**Table 1:** Baseline patient’s characteristics.

Variables	Total (*n* = 32)
Age, years	64.2 ± 9.5
Male	30 (93.8%)
Cause (*n*,%)	
HBV	24 (75.0%)
Others	8 (25.0%)
AST, IU/mL	46.5 [31.5; 66.5]
ALT, IU/mL	21.5 [13.0; 36.5]
T.bil, mg/dL	1.0 ± 0.5
Alb, g/dL	3.7 [3.2; 4.0]
PLT, 103/μL	156.0 ± 81.8
INR	1.1 [ 1.0; 1.2]
Cr, mg/dL	0.7 [0.7; 0.9]
CP class A	32 (100.0%)
AFP, ng/mL	77.9 [6.0; 2381.3]
PIVKA, mAU/mL	1085.2 [220.6; 8170.3]
Tumor size, cm	5.8 [3.0; 10.5]
Multiple intrahepatic HCC	28 (87.5%)
PVTT	22 (68.8%)
Extrahepatic metastasis	17 (53.1%)

AST, aspartate aminotransaminase; ALT, alanine aminotransaminase; T.bil, total bilirubin; Alb, albumin; PLT, platelet; INR, international normalized ratio; CP, Child–Pugh; AFP, alpha-fetoprotein; PVIKA, protein induced by vitamin K absence-II; HCC, hepatocellular carcinoma; PVTT, portal vein tumor thrombus.

**Table 2:** Univariate and multivariable Cox-regression analysis for survival including changes in early cytokine levels before and after 1st atezolizumab/bevacizumab treatment.

Variables	Univariate		Multivariable 1		Multivariable 2		Multivariable 3	
	sHR	*p*-Value	Adjusted sHR (95% CI)	*p*-Value	Adjusted sHR (95% CI)	*p*-Value	Adjusted sHR (95% CI)	*p*-Value
Age (≥65)	0.49	0.200						
Sex (Male)		>0.9						
HBV (vs. others)	1.12	0.800						
Total bilirubin (≥2 mg/dL)	1.40	0.700						
Albumin (≥3 mg/dL)	0.26	0.087						
Platelet (<100 × 10^3^/μL)	2.60	0.066						
CTP class A (vs. class B)	2.76	0.055						
Presence of PVTT	2.98	0.094						
AFP (≥100 ng/mL)	2.35	0.110						
PIVKA-II (≥1000 mAU/mL)	1.54	0.400						
Intrahepatic tumor size (≥5 cm)	6.59	0.014	7.21 (1.49–34.9)	0.014	7.24 (1.54–34.1)	0.012	12.1 (2.03–72.1)	0.006
Multiple intrahepatic tumors	1.06	>0.9						
Presence of extrahepatic metastasis	1.07	>0.9						
Increased IL-10 levels (≥2.15)	0.22	0.011	0.19 (0.05–0.72)	0.014	Not included		Not included	
Increased TNF-α levels (≥2.95)	0.25	0.010	Not included		0.23 (0.08–0.70)	0.009	Not included	
Increased IL-10 and TNF-α levels	0.14	0.011	Not included		Not included		0.07 (0.01–0.46)	0.005

sHR, sub-distribution hazard ratio; CI, confidence interval; AST, aspartate transaminase; ALT, alanine transaminase; CTP, Child–Turcotte–Pugh; PVTT, portal vein tumor thrombosis; AFP, α-fetoprotein; PIVKA-II, protein induced by vitamin K, absence or antagonist-II; IL, interleukin; TNF, tumor necrosis factor.

## Data Availability

Data are not available due to ethical issues.

## Supplementary Materials

The following supporting information can be downloaded at: https://www.mdpi.com/article/10.3390/cancers16203543/s1, Supplementary Figure S1: Overall survival according to changes in cytokine levels before and after atezolizumab/ bevacizumab treatment, including (A) IL-2, (B) IL-6, (C) IL-12, and (D) IFN-γ, based on their median changes in levels. Supplementary Figure S2: Overall survival and progression-free survival at 6 months according to baseline cytokine levels, including (A,B) IL-10, (C,D) IL-17, and (E,F) TNF-α. Supplementary Figure S3: Progression-free survival at 6 months according to changes in cytokine levels before and after atezolizumab/ bevacizumab treatment, including (A) IL-2, (B) IL-6, (C) IL-12, and (D) IFN-γ, based on their median changes in levels.
