# Supplementary material for: An Early Increase in IL-10 and TNF-α Levels Following Atezolizumab Plus Bevacizumab Treatment Predicts Survival in Advanced Hepatocellular Carcinoma Patients: A Prospective Cohort Study
Source: Cancers (Basel). 2024 Oct 21;16(20):3543. doi: 10.3390/cancers16203543 (PMC11506365; doi:10.3390/cancers16203543)
Supplement: Supplementary file 1 [file cancers-16-03543-s001.zip › cancers-3241776-supplementary.pdf]

cancers

MDPI

Supplementary Materials

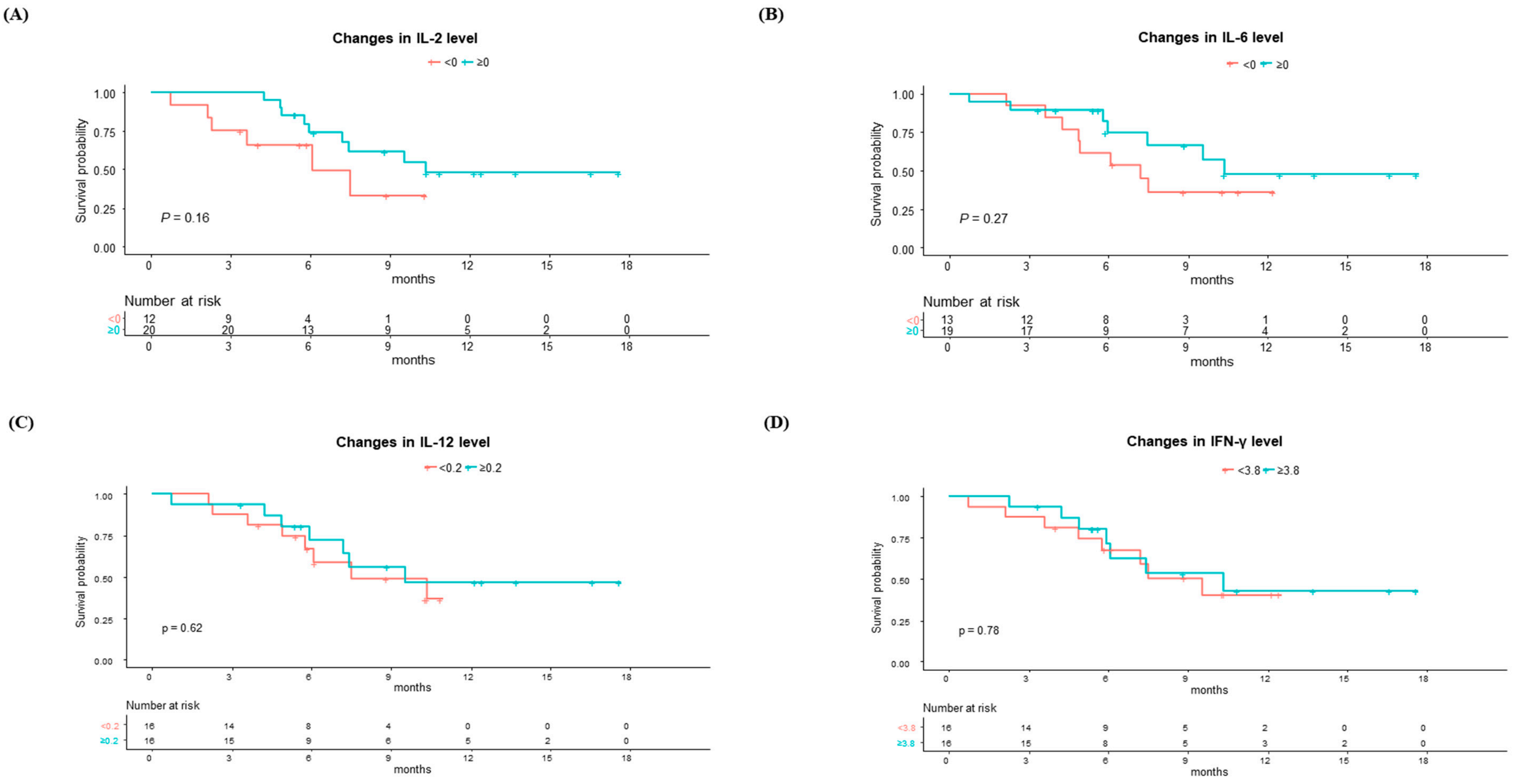

Supplementary Figure S1. Overall survival according to changes in cytokine levels before and after atezolizumab/ bevacizumab treatment, including (A) IL-2, (B) IL-6, (C) IL-12, and (D) IFN-γ, based on their median changes in levels.

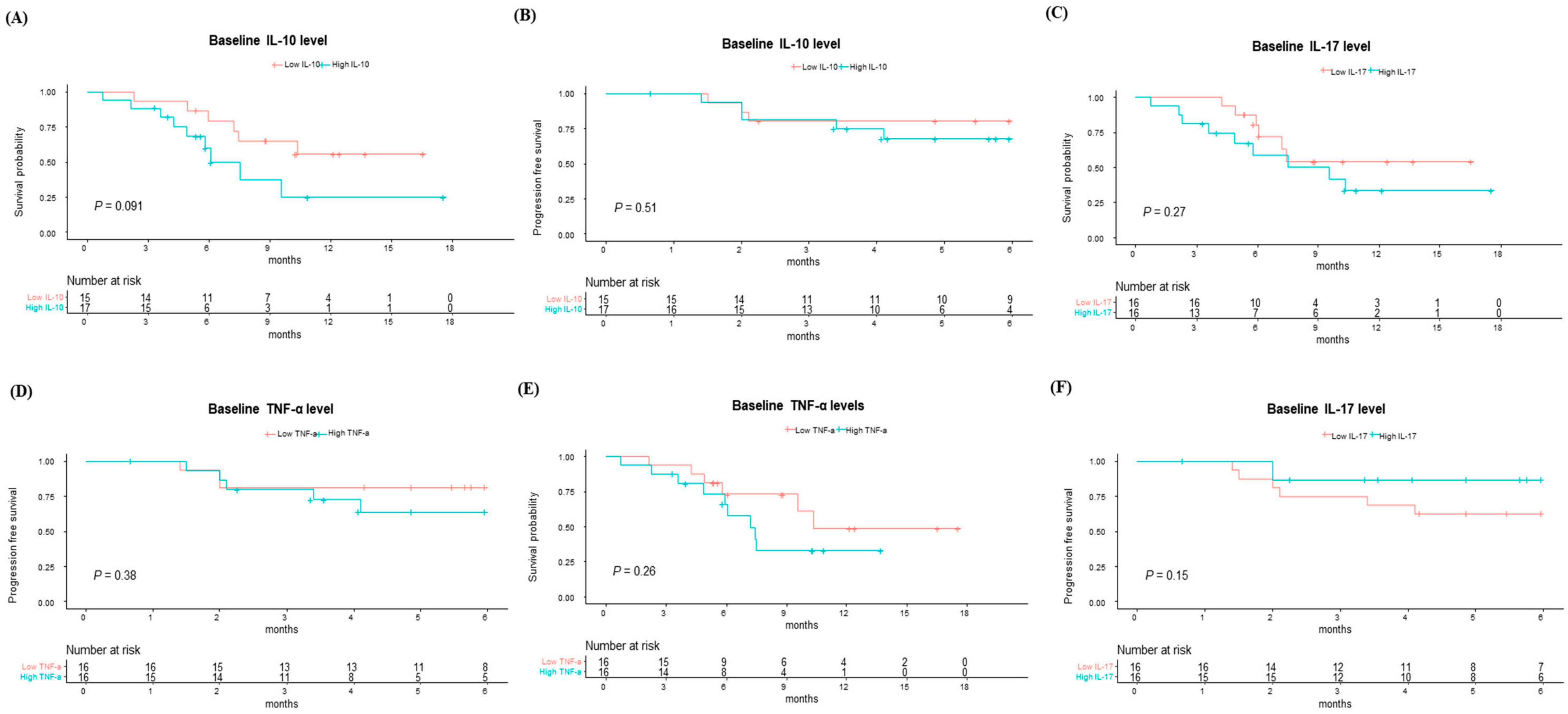

Supplementary Figure S2. Overall survival and Progression-free survival at 6 months according to baseline cytokine levels, including (A,B) IL-10, (C,D) IL-17, and (E,F) TNF-α.

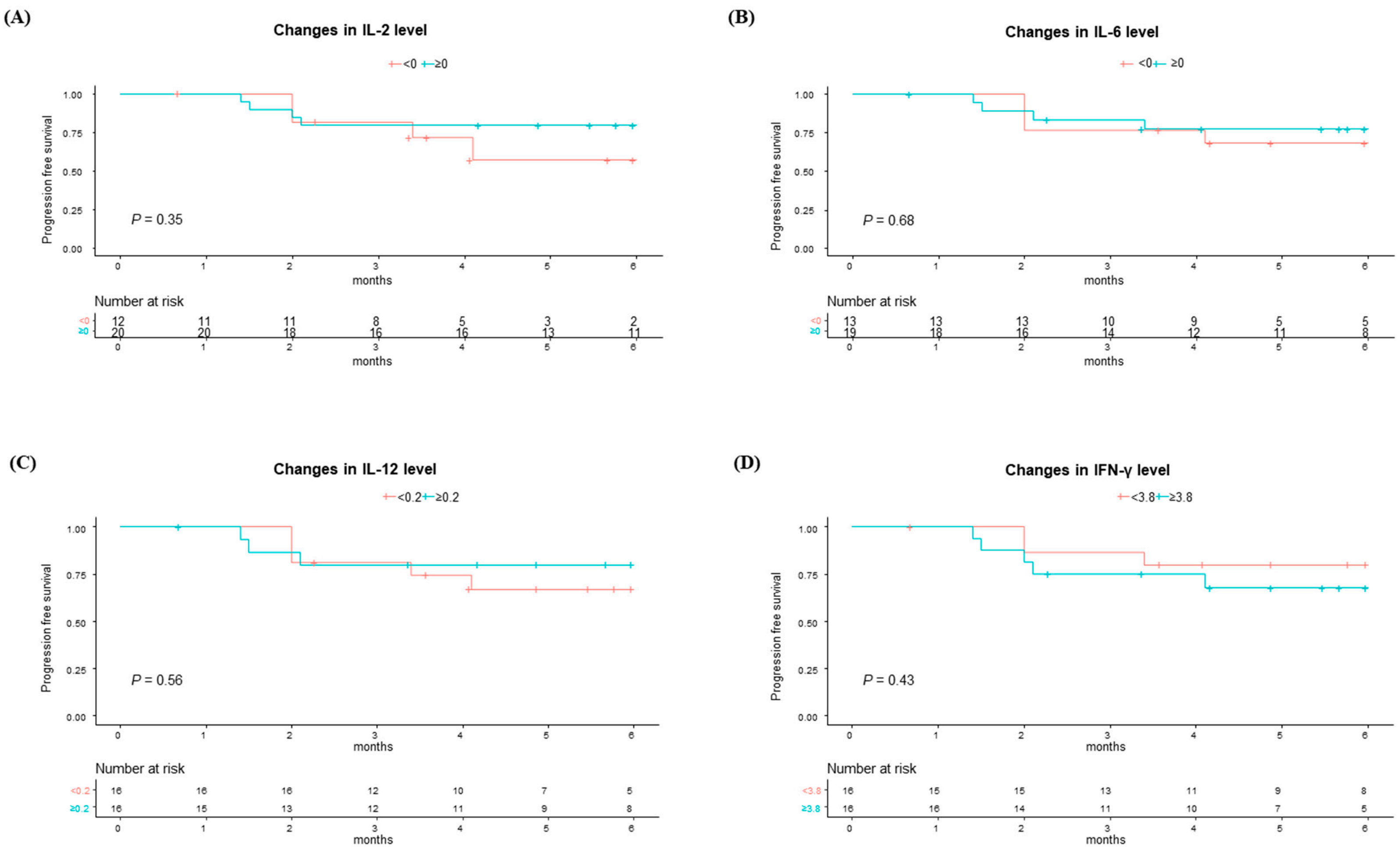

Supplementary Figure S3. Progression-free survival at 6 months according to changes in cytokine levels before and after atezolizumab/ bevacizumab treatment, including (A) IL-2, (B) IL-6, (C) IL-12, and (D) IFN-γ, based on their median changes in levels.
